# Oxidative stress protection and growth promotion activity of *Pseudomonas mercuritolerans* sp. nov., in forage plants under mercury abiotic stress conditions

**DOI:** 10.3389/fmicb.2022.1032901

**Published:** 2022-12-06

**Authors:** Marina Robas Mora, Vanesa M. Fernández Pastrana, Daniel González Reguero, Laura L. Gutiérrez Oliva, Agustín Probanza Lobo, Pedro A. Jiménez Gómez

**Affiliations:** Department of Health and Pharmaceutical Sciences, School of Pharmacy, Universidad San Pablo-CEU, CEU Universities, Madrid, Spain

**Keywords:** heavy metals, PGPB, oxidative stress protection, *Pseudomonas mercuritolerans*, phytoprotection against mercury, mercury contamination

## Abstract

SAICEUPSM^T^ strain was isolated from soils in the mining district of Almadén (Ciudad Real, Spain), subjected to a high concentration of mercury. Using the plant model of lupinus, the strain was inoculated into the rhizosphere of the plant in a soil characterized by a high concentration of mercury (1,710 ppm) from an abandoned dump in the mining district of Almadén (Ciudad Real, Spain). As a control, a soil with a minimum natural concentration of mercury, from a surrounding area, was used. Under greenhouse conditions, the effect that the inoculum of the SAICEUPSM^T^ strain had on the antioxidant capacity of the plant was studied, through the quantification of the enzymatic activity catalase (CAT), ascorbate peroxidase (APX), superoxide dismutase (SOD), and glutathione reductase (GR). Likewise, the capacity of the plant to bioaccumulate mercury in the presence of the inoculum was studied, as well as the effect on the biometric parameters total weight (g), shoot weight (g), root weight (g), shoot length (cm), root length (cm), total number of leaves (N), and total number of secondary roots (No). Finally, in view of the results, the SAICEUPSM^T^ strain was identified from the phenotypic and genotypic point of view (housekeeping genes and complete genome sequencing). The inoculum with the SAICEUPSM^T^ strain in the presence of mercury produced a significant reduction in the enzymatic response to oxidative stress (CAT, APX, and SOD). It can be considered that the strain exerts a phytoprotective effect on the plant. This led to a significant increase in the biometric parameters total plant weight, root weight and the number of leaves under mercury stress, compared to the control without abiotic stress. When analyzing the mercury content of the plant with and without bacterial inoculum, it was found that the incorporation of the SAICEUPSM^T^ strain significantly reduced the uptake of mercury by the plant, while favoring its development in terms of biomass. Given the positive impact of the SAICEUPSM^T^ strain on the integral development of the plant, it was identified, proving to be a Gram negative bacillus, in vitro producer of siderophores, auxins and molecules that inhibit stress precursors. The most represented fatty acids were C16:0 (33.29%), characteristic aggregate 3 (22.80%) comprising C16:1 ω7c and C16: 1ω6c, characteristic aggregate 8 (13.66%) comprising C18:1 ω7c, and C18: 1 cycle ω6c and C 17:0 (11.42%). From the genotypic point of view, the initial identification of the strain based on the *16S rRNA* gene sequence classified it as *Pseudomonas iranensis*. However, genome-wide analysis showed that average nucleotide identity (ANI, 95.47%), DNA-DNA *in silico* hybridization (dDDH, 61.9%), average amino acid identity (AAI, 97.13%), TETRA (0.99%) and intergenic distance (0.04) values were below the established thresholds for differentiation. The results of the genomic analysis together with the differences in the phenotypic characteristics and the phylogenetic and chemotaxonomic analysis support the proposal of the SAICEUPSM^T^ strain as the type strain of a new species for which the name *Pseudomonas mercuritolerans* sp. is proposed. No virulence genes or transmissible resistance mechanisms have been identified, which reveals its safety for agronomic uses, under mercury stress conditions.

## Graphical Abstract

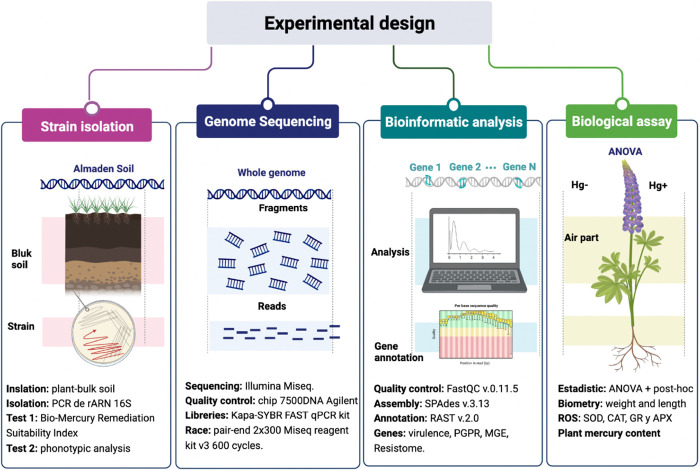

## Introduction

Up to 9,000 tons of mercury are released annually into the atmosphere, water, and soils (Zhang et al., 2021). Mercury is a global pollutant that causes damage to the environment and can affect animals and people by its transmission through the food chain. Therefore, it has the potential to become a public health and environmental threat (Ballabio et al., 2021). For this reason, there is a growing scientific, technical, and social interest in reducing mercury pollution and mitigating its effects. Bioremediation techniques have proven to be an effective and environmentally friendly alternative (Bhatt et al., 2021) as well as useful for the recovery of high land extensions (Dary et al., 2010). *In situ* metal phytostabilization is a phytoremediation technique that uses metal-tolerant plants for the mechanical stabilization of the contaminant, preventing its transport to other environments by leaching or air transport. In addition, it reduces the accumulation of pollutants in biological systems, such as plants. Mercury is a heavy metal that has no biological role in plants or animals. For this reason, it tends to bioaccumulate, replacing other metabolically active metals in the body and triggering numerous diseases (Ballabio et al., 2021). Phytostabilization therefore sequesters contaminants in the soil environment in a more cost-effective way, especially in the case of extensive contamination. For this purpose, the use of fast-growing forage plants, such as the *Lupinus* genus, may be useful.

Phytostabilization process can be improved with the use of microorganisms with the ability to degrade contaminants and/or with the ability to promote plant growth (PGPB, plant growth promoting bacteria) under stress conditions (Kuiper et al., 2004; Azaroual et al., 2022). Traditionally, PGPB were used primarily to help plants absorb nutrients from the environment or to prevent plant diseases, in both cases by promoting their development.

Despite the natural potential of plants to remove heavy metals from the soil, phytoremediation is yet to become a commercially available technology. For this reason, it seems interesting to investigate beneficial plant-microorganism associations that improve the efficiency of the phytoremediation process. The *Pseudomonas* genus is varied and has great versatility. Many of its species can colonize different niches, due to their metabolic capacity and their ease of adaptation to different conditions (Bravakos et al., 2021). The association with plants is enhanced by the secretion of phytohormones (auxins, gibberellins, etc.), secondary metabolites (flavonoids) and enzymes (aminocyclopropane-1-carboxylate, phenylalanine ammonia-lyase) as well as siderophores, nitrogen fixation, sulfate solubilization, antibiotic production, induced systemic resistance (Sah et al., 2021; Consentino et al., 2022; Mellidou and Karamanoli, 2022), and phytopathogens control (Zhang et al., 2022). SAICEUPSM^T^ was isolated from the rhizosphere of a *Medicago sativa* plant native to the Almadén mining district. Specifically, it was isolated from a dump slope, where the mercury concentration was 1,071 ppm (Millán et al., 2007). Due to its ability to promote plant growth under conditions of abiotic stress, the strain was identified, and it was verified that both bioinformatics and phenotypic analysis suggest that it may be a new strain, reason why *Pseudomonas mercuritolerans* is proposed as its new taxonomic classification.

The aim of this work is the evaluation of the *in vivo* mercury phytostabilization potential under greenhouse conditions of *Lupinus albus* var., Orden Dorado plants in association with mercury resistant SAICEUPSM^T^ strain in soil substrates from the Almadén mining district (Ciudad Real, Spain).

## Results

### Isolation, identification, and phylogenetic analysis

Strain SAICEUPSM^T^ isolated from the rhizosphere of a *Medicago sativa* plant native to the Almadén mining district was classed as a Gram negative rod-shaped bacterium forming translucent brown colonies that intensify their hue after 72 h, ≈ 1 mm ∅, with smooth borders, and creamy texture. It grows in nutritive agar at 28°C, with aerobic metabolism, oxidase catalase positive, non-endospore-forming. Its size is 624.1–778.7 μm wide and 1.679–2.489 μm long (Supplementary Figure 1). These characteristics identify SAICEUPSM^T^ as belonging to the *Pseudomonas* genus.

The similarity analysis using the *16S rRNA* gene showed a value of 96.60% against *P. iranensis* SWRI54. All other strains analyzed showed lower similarity indices (Table 1).

**TABLE 1:** Similarity of 16S rRNA with the 13 closest taxa and the SAICEUPSM^T^ strain.

Subject	Type strain (T)	Adhesion	Identity (%)
*Pseudomonas iranensis*	SWRI54	CP077092.1	96.60%
*Pseudomonas allokribbensis*	IzPS23	CP062252.1	90.65%
*Pseudomonas hamedanensis*	SWRI65	CP077091.1	90.08%
*Pseudomonas kribbensis*	46-2	CP029608.1	89.52%
*Pseudomonas tensinigenes*	ZA 5.3	CP077089.1	89.39%
*Pseudomonas gozinkensis*	IzPS32d	CP062253.1	88.81%
*Pseudomonas glycinae*	MS589	CP014205.2	87.96%
*Pseudomonas zeae*	OE 48.2	CP077090.1	87.82%
*Pseudomonas monsensis*	PGSB 8459	CP077087.1	87.27%
*Pseudomonas azerbaijanoriens*	SWRI123	CP077078.1	85.19%
*Pseudomonas eucalypticola*	NP-1	CP056030.1	84.60%
*Pseudomonas mandelii*	LMG26867	LT629796.1	84.74%
*Pseudomonas prosekii*	LMG26867	LT629762.1	84.62%

**FIGURE 1:**
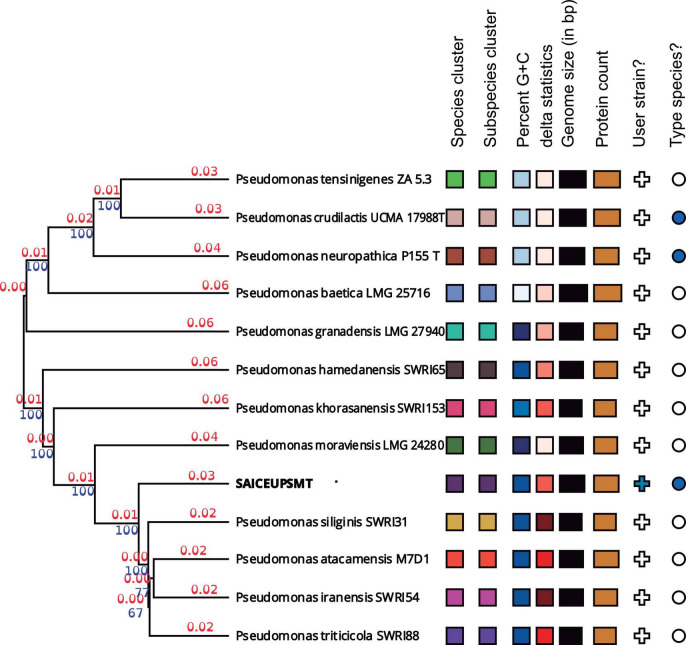
Phylogenetic tree from the complete genome of the species of the genus *Pseudomonas*. Numerical values indicate the relative distance between the analyzed species. The branch lengths are scaled in terms of Genome Blast Distance Phylogeny (GBDP) d5. The blue numbers are GBDP pseudo-bootstrap support values >60% from 100 replications, with an average branch support of 55.2%. The branch length values (in red) represent the evolutionary time between two nodes. Unit: substitutions per sequence site. The tree was rooted at the midpoint.

Whole genome sequence (WGS) analysis revealed that average nucleotides identity value (ANI) value against *P. iranensis* SWRI54 was 95.34%. In the same way, the average amino acids identity (AAI) offered a value of 97.04%. For this reason, it cannot be concluded that the SAICEUPSM^T^ strain belongs to *P. iranensis* SWRI54. According to the of the CLSI identification criteria (Clinical and Laboratory Standards Institute) strain SAICEUPSM^T^ could be a new species within the *Pseudomonas* genus. The phylogenetic activity of Genome Blast Distance Phylogeny (GBDP) (Figure 1) positioned SAICEUPSM^T^ within the cluster of *P. siliginis* SWRI31, *P. atacamensis* M7D1, *P. iranensis* SWRI54 and *P. triticicola* SWRI88. SAICEUPSM^T^ is segregated from the species in its cluster. The threshold used to discriminate nearby species is between 95–96% for ANI and 70% for DNA-DNA hybridization *in silico* (dDDH) (Richter and Rosselló-Móra, 2009). The analysis of dDDH shows that the highest value for SAICEUPSM^T^ is below this threshold (Table 2). The ANI analysis was 95.42%. These data confirm that the SAICEUPSM^T^ strain does not belong to any of these species (Supplementary Table 1: Calculated ANIb values, Supplementary Table 2: Calculated Tetra values, and Supplementary Table 3: Intergenomic distance).

**TABLE 2:** DNA-DNA hybridization *in silico* (dDDH), confidence interval (CI), and GC percentage difference between SAICEUPSM^T^ and *Pseudomonas* spp. closely related.

Subject	Assembly accession	dDDH	CI	Diff GC mol %
*Pseudomonas atacamensis* M7D1	GCA_004801935	61.7	[58.9–64.5]	0.01
*Pseudomonas iranensis* SWRI54	GCA_014268585	61.5	[58.6–64.3]	0.01
*Pseudomonas triticicola* SWRI88	GCA_019145375	60.8	[57.9–63.6]	0.10
*Pseudomonas siliginis* SWRI31	GCF_019145195	59.7	[56.8–62.4]	0.09
*Pseudomonas moraviensis* LMG 24280	GCF_900105805	46.8	[44.2–49.4]	0.28
*Pseudomonas khorasanensis* SWRI153	GCA_014268505	37.6	[35.2–40.1]	0.17
*Pseudomonas hamedanensis* SWRI65	GCA_014268595	36.8	[34.3–39.3]	0.11
*Pseudomonas granadensis* LMG 27940	GCA_900105485	34.0	[31.5–36.5]	0.27
*Pseudomonas tensinigenes* ZA 5.3	GCA_014268445	33.6	[31.2–36.1]	0.71
*Pseudomonas crudilactis* UCMA 17988T	GCA_017973755	33.5	[31.1–36.0]	0.75
*Pseudomonas neuropathica* P155 T	GCF_015461835	33.5	[31.1–36.0]	0.66
*Pseudomonas baetica* LMG 25716	GCA_002813455	33.2	[30.8–35.8]	1.13

Formula d4 (a.k.a. GGDC formula 2): sum of all identities found in HSPs divided by overall HSP length.

### Phenotypic description and plant growth promotion activities characterization

This strain is a siderophores producer and presents the ability to degrade ethylene precursor 1-aminocyclopropane-1-carboxylate deaminase (ACC) *via* enzyme ACC deaminase (ACCd). Additionally, produces the auxin class phytohormone Indole-3-acetic acid (IAA, 7.72 μg.ml^–1^). Metabolizes citrate, L-Proline aryl amidase and hydrolyzes gelatin, beta hemolytic, and urea transformer. It was negative for the motility, production of acetoin, fermentation of dulcitol, deamination of phenylalanine, production of indole, hydrogen sulfide, decarboxylation of ornithine, lysine, arginine, sucrose, xylose, maltose, mannitol, citrate, and lactose. Table 3 shows different phenotypic characteristics compared to the species with greater phylogenetic proximity.

**TABLE 3:** Comparison of the phenotype of SAICEUPSM^T^ with its closest species according to its DNA-DNA hybridization *in silico* (dDDH).

	1	2	3	4	5	6	7	8	9	10	11	12
Fluorescent pigments in King B medium (fluorescein)	+	+	−	+	−	+	+	−	+	+	+	+
Growth at 37°C	+	NA	+	−	−	−	NA	+	NA		+	+
Growth in nutritive agar + NaCl 6%	−	NA	−	/	+	+	NA	−	NA	+	+	+
Nitrate reduction	−	−	−	−	−	+	NA	−	NA	NA	+	−
Citrate utilization	+	+	+	+	NA	+	NA	+	NA	NA	NA	+
Gelatin hydrolysis	−	+	+	+	−	+	+	+	+	+	−	
D-Glucose (oxidation)	+	+	+	+	+	+	+	+	+	+	+	+
L-arabinose (oxidation)	+	+	+	−	+	+	+	+	+	+	+	−
D-galactose (oxidation)	−	+	+	+	+	+	+	+	/	/	+	−
Trehalose (oxidation)	+	+	+	−	−	NA	NA	+	NA	NA	NA	−

Taxons: 1: *Pseudomonas atacamensis* M7D1; 2: *P. moraviensis* LMG24280; 3: *P. granadensis* LMG 27940; 4: *P. baetica* LMG 25716; 5: *P. crudilactis* UCMA 17988T; 6: *P. koreensis* LMG21318; 7: *P. atagonensis* PS14; 8: *P. iridis* P42; 9: *P. allokribbensis* LMG31525T; 10: *P. gozinkensis* LMG31526; 11: *P. laurylsulfativorans* AP3_22; 12: SAICEUPSM^T^; +: positive; −: negative; /: weak; NA: data is not available.

The bacterium can grow at concentrations between 0 and 6% (p/v) of NaCl, the optimal pH is between 5.5 and 8.0 and within the temperature range of 4 to 37°C, being 28°C its optimal growth temperature. The minimum inhibitory concentrations (MIC) against different antibiotics are shown in Supplementary Table 4.

Regarding the resistance of the strain against heavy metals, quantified by calculating the minimum bactericidal concentration (MBC), SAICEUPSM^T^ strain was highly resistant to mercury (140 ppm), cupper (400 ppm), chrome (800 ppm), and nickel (400 ppm). In contrast, the cadmium tolerance capacity was 12.5 ppm.

### Chemotaxonomic analysis

The analysis by mass spectrophotometry measured by matrix-assisted laser desorption/ionization–time of flight (MALDI-TOF) generated a list of 10 peaks based on their intensity, which allowed a peptide fingerprint and its comparison with the database (Supplementary Figure 2). The analysis of this profile showed a homologation between SAICEUPSM^T^ with *P. fluorescens*.

The analysis of fatty acids showed the best represented molecules: C16:0 (35.59%), Sum in Feature 3 (18.21%), C17:0 cyclo (16.73%), and Sum in Feature 8 (10.78%). The profile described by SAICEUPSM^T^ does not allow the identification of the strain with any nearby species (Table 4).

**TABLE 4:** Fatty acid improve titration.

	1	2	3	4	5	6	7	8
**Saturated fatty acid**								
C_10:0_	–	–	0.1	–	–	–	–	–
C_12:0_	2.40	+	1.4	1.6	2.26	1.80	1.99	4.09
C_14:0_	0.53	+	0.6	–	–	+	+	–
C_16:0_	35.59	24.67	33.7	32.8	36.09	31.93	32.04	–
C_17:0_	0.56	–	–	–	–	–	–	–
C_18:0_	0.76	–	0.4	+	–	+	+	–
**Branched fatty acid**								
C_17:0_ cyclo	16.73	+	2.8	11.5	7.48	16.13	17.69	1.31
C_18:1_ω 7c	–	9.53	–	–	–	–	–	11.22
C_19:0_ cyclo 8cω	0.89	–	–	–	–	+	+	–
**Hydroxy fatty acid**								
C_12:0_ 2-OH	5.24	10.27	5.2	5.3	5.36	5.14	4.90	3.17
C_10:0_ 3-OH	3.23	+	4.0	3.2	4.16	4.08	4.24	3.89
C_12:0_ 3-OH	4.65	+	–	4.5	4.28	5.40	5.03	4.22
C_12:1_ 3-OH	–	–	–	–	–	–	–	1.86
**Summed features**								
2	–	–	–	–	–	–	–	10.9
3	18.21		36.2	27.2	30.66	19.82	18.27	38.81
8	10.78		11.3	10.7	10.53	10.92	10.69	

Taxons: 1: *Pseudomonas atacamensis* M7D1; 2: *P. moraviensis* LMG24280; 3: *P. granadensis* LMG 27940; 4: *P. baetica* LMG 25716; 5: *P. crudilactis* UCMA 17988T; 6: *P. koreensis* LMG21318; 7: *P. atagonensis* PS14; 8: *P. iridis* P42; 9: *P. allokribbensis* LMG31525^T^; 10: *P. gozinkensis* LMG31526; 11: *P. laurylsulfativorans* AP3_22; 12: SAICEU98^T^; –: not detected; +: detected in small unspecified quantities.

### Genome features

The SAICEUPSM^T^ genome was formed from 192 contigs, with a genome length of 6,312,264 bp. The GC content was 60.07% mol (Supplementary Figure 3A). A total of 5,522 CDS were identified and assigned to 27 subsystems through the Rapid using Subsystem Technology (RAST) SEED viewer (Supplementary Table 5 and Supplementary Figure 3B), achieving the score of 42% (2337) and through the KEGG analysis 44%. The most represented subsystems were amino acids and derivatives (481), carbohydrates (262), protein metabolism (220), cofactors, vitamins, protein, and pigments (206). The genome sequence project was deposited in NCBI (Bioproject PRJNA847155; Biosample SAMN28920853).

KofamKOALA analyses show that almost all the bacteria’s major metabolic pathways were found in SAICEUPSM^T^ genome (Supplementary Figure 4A and Supplementary Table 6). An in-depth analysis of the mechanisms that could support the phenotypic result of resistance to mercury revealed that SAICEUPSM^T^ had genes from the mercury resistance operon, finding transport genes (mercuric transport protein, *merT* and mercuric transport protein, *merC*) and reduction genes (Mercuric ion reductase, *merA* and Organomercurial lyase, *merB*) (Supplementary Figure 4B). Likewise, the existence of genes that confer resistance to other heavy metals (cadmium, cupper, chrome, nickel), was analyzed to reinforce the phenotypic observations. The results of the whole gene collection can be consulted extensively in Supplementary Table 7.

The list of genes that code for mechanisms of antibiotic resistance are shown in Supplementary Table 8. Likewise, Supplementary Tables 9, 10 show the genes involved in the main virulence factors: biosynthesis of flagella proteins, adhesion motility, endotoxin, type IV pili, adherence, contraction rate, ion uptake, antiphagocytosis, and secretion systems Type I, Type II, Type III, and Type VI. These operons are incomplete, which is why the strain is not able to express them functionally. The software of the Center for Genomic Epidemiology predicted that the organism does not present a risk of pathogenesis.

In Supplementary Table 11, genes associated with direct and indirect mechanisms of plant growth promoters in SAICEUPSM^T^ are collected.

### Plant growth promotion

**FIGURE 2:**
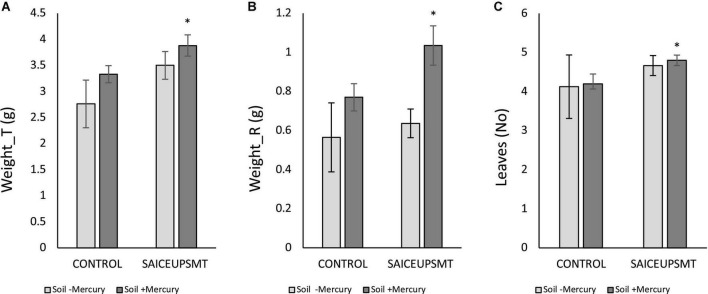
Results of the least significance difference (LSD) analysis of the biometric parameters of plants grown in soils with mercury. **(A)** Total weight, **(B)** root weight, and **(C)** total number of leaves. The bars indicate a standard error. *p-value ≤ 0.05. s/u, no units.

The ANOVA analysis of the biometric variables of those plants inoculated with SAICEUPSM^T^, revealed the existence of significant differences (*p-*value < 0.05) in the parameters total weight (Weigth_T, Figure 2A), root weight (Weight_R, Figure 2B), and number of leaves (Leaves, Figure 2C) compared to the controls. The SAICEUPSM^T^ strain significantly contributed to a higher plant weight, as well as a greater development of the root with respect to the control.

### Phytoprotection

**FIGURE 3:**
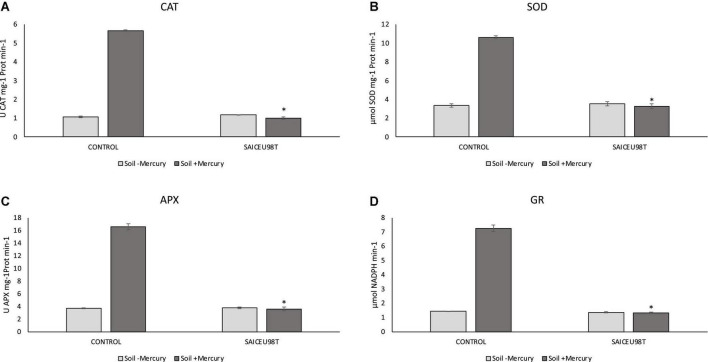
Results of Kruskal–Wallis for enzymatic content: **(A)** catalase (CAT), **(B)** superoxide dismutase (SOD), **(C)** ascorbate peroxidase (APX), and **(D)** glutathione reductase (GR). The bars indicate the standard error. *p-value ≤ 0.001.

To verify the potential oxidative stress protection effect SAICEUPSM^T^ strain under mercury stress conditions, the enzymatic response of *Lupinus albus* grown in soil with a high concentration of the heavy metal was evaluated. Figure 3 shows quantification of catalase (CAT), superoxide dismutase (SOD), ascorbate peroxidase (APX), and glutathione reductase (GR) enzymes. Incorporating the strain into the root promotes a significant reduction in enzymatic response.

### Mercury accumulation in plant

To evaluate the potential mercury phytoprotective capacity of SAICEUPSM^T^, the physiological content of this heavy metal in the plant was analyzed (Table 5). It is observed that *Lupinus albus* tends to bioaccumulate mercury mainly at the root level. This accumulation is not statistically different in inoculated plants. The incorporation of SAICEUPSM^T^, promoted a significant decrease in the accumulation of mercury in the shoot of the plant, respect the control.

**TABLE 5:** Mercury accumulation in plant, tested in soils with high mercury concentration.

Substrate	Inoculum	Shoot (μg.g^–1^)	Root (μg.g^–1^)
Soil + [mercury]	–	0.21 ± 0.02	10.06 ± 0.15
Soil + [mercury]	SAICEUPSM^T^	0.13 ± 0.01*	10.04 ± 0.14

*p-value ≤ 0.001.

## Discussion

*Lupinus albus* model has shown to be a plant with high tolerance to abiotic stress (Fumagalli et al., 2014) as well as having an interesting phytoextraction capacity (Zornoza et al., 2010; Godínez-Méndez et al., 2021; Quiñones et al., 2021). However, mercury is also known to induce physiological and metabolic alterations in plants, as the enzymatic response against oxidative stress (Zelles, 1999; Christakis et al., 2021; Çavus̨oğlu et al., 2022) and decreased plant growth. Conversely, the effect of PGPB inoculation may minimize these effects on plants (Heidari and Golpayegani, 2012; Morcillo and Manzanera, 2021) in mercury-contaminated substrates (Pirzadah et al., 2018). The *Pseudomonas* genus, precisely, has shown in previous works its important role as PGPB in stress conditions (Sandhya et al., 2010). In those plants inoculated with SAICEUPSM^T^, the biometric parameters total weight, root weight and number of leaves were significantly higher in soils with high mercury concentration, compared to the same growth conditions, in the absence of PGPB inoculum. The identification of genes coding for plant growth promotion (PGP) activities explains the phenotype of the bacterium. This fact has already been described in other *Pseudomonas* species (Chellaiah, 2018; Kang et al., 2020).

From the results of this work, we confirm that, inoculation with SAICEUPSM^T^ strain under mercury stress produces a significant reduction in the enzymatic response to oxidative stress (CAT, APX, and SOD). SOD enzyme catalyzes the transformation of singlet oxygen to H_2_O_2_, with CAT and APX enzymes being responsible for eliminating H_2_O_2_ by transforming it into H_2_O and O_2_ (Gill and Tuteja, 2010). Similarly, GR is involved in reducing glutathione disulfide (GSSG) to glutathione (GSH) with NADPH expenditure. GSH plays a very important role in the redox regulation of the cell cycle and in the defense mechanisms against oxidative stress (Sánchez-Fernández et al., 1997). Plants grown in mercury contaminated soils and inoculated with SAICEUPSM^T^ have also significantly lower GR production. Root colonization by SAICEUPSM^T^ produces a phytoprotection effect against mercury stress by inducing a better antioxidant enzymatic response, compared to the non-inoculated controls.

Plants of different species, like *Lupinus albus*, can accumulate mercury in different tissues, but the absorption mechanism is still unknown (Zornoza et al., 2010; Quiñones et al., 2021; Robas et al., 2021). This fact can induce a reduction in the production of plant biomass. On the contrary, plants inoculated with SAICEUPSM^T^ can increase the weight and number of leaves of plants, even in substrates with high concentration of mercury. The incorporation of SAICEUPSM^T^ significantly reduces the absorption of mercury by the plant, while favoring its development in terms of biomass. That is why we can speak of a phytoprotective effect, which can be associated with the transformation of mercury carried out by the bacteria in the root environment, due to the expression of the genes involved in the metabolism of this heavy metal *mer* (operon). This fact is key for future agronomic applications of the strain, since the improvement in production can be significant, even under conditions of high concentration of mercury, and bioremediation processes. All of this, under safe (environmental and health) conditions since the innocuousness of the SAICEUPSM^T^ strain has been demonstrated as it lacks functional virulence factors and transmissible or clinically relevant resistance genes. For all the above, the SAICEUPSM^T^ strain is postulated as a new taxon not described to date, called *P. mercuritolerans*, with potential use in improving plant yield under abiotic stress conditions and in bioremediation processes of heavy metals, such as mercury.

The growing soil heavy metal pollution in natural environments has increased scientific interest in finding alternatives for the decontamination of these ecosystems. To this end, the participation of PGPB can contribute effectively, both directly on the soil and indirectly, by contributing to the phytoprotection of plants (Singh et al., 2019). The *Pseudomonas* genus can grow and develop under unfavorable conditions, metabolize a wide variety of organic and inorganic compounds, as well as reduce and volatilize heavy metals (Beltrán-Pineda and Gómez-Rodríguez, 2016). SAICEUPSM^T^ strain has functional genes related to the reduction and volatilization of mercury, resistance to copper, cobalt, zinc, cadmium, as well as siderophores production capacity that help improve tolerance or transform ions of toxic metals, minimizing their toxic effects (Singh and Hiranmai, 2021), which could justify the high resistance values obtained in laboratory tests. There are few bibliographical references that review the tolerance of different species of the *Pseudomonas* genus to mercury. However, the MBC values found in this work highlight the unique resistance of SAICEUPSM^T^ to said heavy metal. Specifically, phenotypically, the strain ceased to be viable at a concentration of 140 ppm (516.6 μm HgCl_2_). Sahu et al. (2016) isolated a strain that they considered to be highly tolerant to mercury isolated from an industrial effluent, capable of withstanding up to 200 μm HgCl_2_ (i.e., almost 2.5 times less than SAICEUPSM^T^). In this same sense, Zhang et al. (2012) isolated from a marine sample, a strain of *P. putida* with a tolerance to mercury like the previous authors, of 280 μm, also a much lower value than SAICEUPSM^T^. This makes it an excellent candidate for the design of subsequent bioremediation processes for soils contaminated by mercury. The capacity of the strain to resist other heavy metals remains uniquely high, well above values found by other authors (Matyar et al., 2010; Singh et al., 2010) in the *Pseudomonas* genus.

Phenotypic and genotypic characterization techniques allow a multiphase approach of the strains with biotechnological interest. SAICEUPSM^T^ activity and the set of metabolic tests allowed it to be differentiated from nearby species, such as *P. atacamensis* M7D1. Fatty acid analysis has also been commonly used to identify species and describe new taxa (Cody et al., 2015; Heir et al., 2021). Unfortunately, this method does not always allow accurate identification. Despite the existence of undescribed profiles, the lack of homology makes us think of the possibility of the discovery of a new taxon.

Initially, the analysis of *16S rRNA*, a basic tool for classifying bacterial species, showed a similarity of 96.60% against *P. iranensis* strain SWRI54. This value is below the limits for the demarcation of species, commonly accepted (98.65%). Although the comparison of housekeeping genes is very useful for the identification of bacterial species usually isolated in the clinical field, this marker does not allow discrimination in many isolated environmental strains (Saha et al., 2019). Fortunately, the use of bioinformatics tools for the analysis of the whole genome sequence and its content has been revealed as a useful tool for discrimination and taxonomic ordering (Gutierrez-Albanchez et al., 2021).

The analysis of the total genome size of SAICEUPSM^T^ was comparable with that of its closest relatives, all of them belonging to the *Pseudomonas* genus. Overall genome sequence comparisons revealed an ANI value between *P. atacamensis* M7D1 and SAICEUPSM^T^ strain of 95.46% and a dDDH hybridization value of 61.7%. Both values are significantly below the recommended species demarcation values (8.7 and 70%, respectively) (Chun et al., 2018). This suggests that SAICEUPSM^T^ does not belong to any described species.

The most common mechanism of resistance to mercury in Gram negative bacteria consists of the expression and orderly participation of *merA*, *merB*, *merT*, and *merC* genes included in the *mer* operon. *merA*, has the ability to reduce Hg(II) to Hg(0), being this last one volatile. When the substrate is an organomercurial compound, such as methyl-Hg, it is *merB* that catalyzes the protonolysis cleavage of the C-Hg bond, reducing the Me-Hg to methane (CH_4_), and Hg(II) (Christakis et al., 2021). The presence in SAICEUPSM^T^ genome of all these genes explains their mercury resilience and tolerance against. The fact that this operon does not have a repressor could also explain its high value of MCB which allows to postulate its bioremediatory application.

Antibiotics resistance genes found in SAICEUPSM^T^ are not transmissible and are common in *Pseudomonas* genus (Heir et al., 2021). On the other hand, genes involved in virulence mechanisms seem to lack functional capacity.

For all the above, we confirm that, inoculation with SAICEUPSM^T^ strain produces under mercury stress, a significant reduction in the enzymatic response to oxidative stress, significantly promotes plant fitness (increasing total weight, root weight, and number of leaves) and prevents the plant from absorbing mercury from its environment, significantly decreasing its translocation into the shoot. Therefore, SAICEUPSM^T^ strain is postulated as a new taxon not described to date, called *P. mercuritolerans*, with potential use in improving plant yield under abiotic stress conditions and in heavy metals bioremediation processes, such as mercury.

### Description of *Pseudomonas mercuritolerans* sp. nov.

*Pseudomonas mercuritolerans* (adj.) refers to the strain’s tolerance to mercury. It was isolated in 2014 from an autochthonous *Medicago sativa* rhizosphere grown in soils highly contaminated by mercury in the mining district of Almadén (Ciudad Real, Spain). Cells are Gram negative, aerobic, rod-shaped, ranging from 624.1−778.7μm width to 1,679−2,489μm length, non-endospore forming and non-motile. The colonies grow in nutritive agar at an OGT of 28°C forming translucent, round, white-beige colonies. Growth occurs from 4 to 37°C in 24 h. It grows in a pH range of 5.5–8.0 and with a NaCl concentration of 0 to 6%. The optimal temperature, pH, and growth salinity for SAICEUPSM^T^ are 28°C, pH 7 and 0% salinity. It can’t ferment carbohydrates, but it can use urea as a nitrogen source and citrate as the only carbon source; it is catalase and oxidase positive. It produces siderophores (pyoverdine) and can hydrolyze gelatin. The main fatty acids (>81.31% of total fatty acids) were C16:0 (35.59%), sum in feature 3 (18.21%), C17:0 cyclo (16.73%), and sum in Feature 8 (10.78%). The GC genomic DNA content of the type strain is 61.10 mole%. The whole genome sequence has been deposited in the NCBI Bioproject (PRJNA access number 847155), BioSample (SAMN 28920853).

## Materials and methods

### Legume plant for phytoremediation

*Lupinus albus* var., Orden Dorado plants were used for the *in vivo* phytoremediation experiment. Seeds were provided by the seed bank of the Center for Scientific and Technological Research of Extremadura (Spain).

### Bacteria for phytoremediation

SAICEUPSM^T^ was selected from a larger set of isolates for its special promotion of plant growth under mercury stress conditions.

#### Plant growth promotion activities

To determine the *in vitro* production capacity of auxins (3-indoleacetic acid, IAA), a colorimetric technique was used with Van Urk Salkowski’s reagent (Ehmann, 1977). The bacterium was grown in LB medium (Texas, USA) at 28°C for 4 days, in shaking conditions. The liquid medium was then centrifuged. In total, 1 ml of the supernatant was mixed with 2 ml of Van Urk Salkowski reagent (2% FeCl_3_ in 35% HClO_4_ solution) and kept in darkness. Optical density (OD) was measured at 530 nm after 30 min and 120 min. Results were quantified in ppm (μg.ml^–1^). Glick protocol (Glick, 1995) was followed to differentiate the degradation of ACC by the action of the enzyme ACCd of bacteria that could fix nitrogen. The culture medium contained 1.8% Bacto-Agar (Difco Laboratories, Detroit, MI, USA), low in nitrogen content, supplemented with ACC (30 mmol). The plaques were then inoculated and cultured for 3 days at 28°C, monitoring growth daily. The results were qualitatively evaluated (presence/absence of ACCd enzyme). The production of siderophores was quantified using chrome Azurol S (CAS) agar, described by Alexander and Zuberer (1991) the interpretation was based on the quantitative analysis of the production of siderophores, manifested by the appearance of a halo around the bacteria colonies after 72 h of incubation at 28°C. The ability to solubilize phosphates was tested following the protocol described by De Freitas et al. (1997). Tricalcium phosphate agar (TPM) medium was used (Nautiyal, 1999), with a final pH set to 7 with 1 mole.l^–1^ with HCl. After inoculation, the plaques were incubated at 28°C for 72 h. The inorganic phosphates solubilizer colonies showed clarification halos that were evaluated qualitatively (presence/absence). All PGPB activities were analyzed in triplicate.

#### Determination of mercury and other heavy metals minimum bactericidal concentration

Mercury, cadmium, copper, chrome, and nickel MBC was evaluated using Müller Hinton agar (Pronadisa^®^, Madrid, Spain), supplemented with different concentrations of heavy metal salts [HgCl_2_, CdSO_4_, CuSO_4_, Cr_2_(SO_4_)_3_, NiSO_4_]: 800, 400, 350, 200, 175, 150, 100, 87.5, 75, 50, 43.75, and 25 μg.ml^–1^. MBC was determined to be the lowest concentration of heavy metals salts capable of visually inhibiting >99.9% of bacterial growth after 24 h incubation. All assays were carried out in triplicate.

### Substrates and setup of the experiment trays

Two types of substrates were used for the tests. The first of them was a soil with a high concentration of mercury, coming from a mining product dump (called Cerro de los Buitrones) in the Almadén mining district (38° 77′ 35′′ N; 4° 85′ 07′′ O, Ciudad Real, Spain). The total mercury concentration in this first substrate is 1,710 ppm (Millán et al., 2007).

The second substrate, used as a control, was a soil with a minimum natural concentration of mercury, from the area furthest, called Fuente del Jardinillo (38° 76′ 01′′ N; 4° 76′ 79′′ O, Ciudad Real, Spain). The use of this substrate instead of one devoid of mercury was done to guarantee the physical-chemical homogeneity of the sample. In both cases, rhizosphere-free soil samples were taken (≈50 kg per soil) from the surface down to 30–35 cm. They were transported under refrigerated conditions (4°C) to the laboratory, where they were stored (4°C) until the assay (<24 h after sampling). Both soils were sieved to guarantee the elimination of the most voluminous fractions and to homogenize the granulometry in each test and replicas.

Four sterile forest trays were used (Plásticos Solanas S.L., Zaragoza, Spain), each composed of twelve alveoli, with a capacity of 300 cm^3^, with a light of 5.3 × 5.3 cm. Four pre-germinated *Lupinus albus* seeds (2.0 ± 0.5 cm emerged radicle) were sown in each alveolus.

### Inoculant preparation

SAICEUPSM^T^ strain was incubated in nutritive agar (Pronadisa^®^, Madrid, Spain) supplemented with 50 ppm of HgCl_2_ (24 h, 25°C). A bacterial suspension was prepared in 0.45% saline solution and adjusted to 0.5 McFarland (OD 10^8^ cfu.ml^–1^). It was intended to avoid an increase in salinity, which could be aggravated by the incorporation of mercury salts, and which could compromise the correct development of seedlings. Each seed was inoculated with 1 ml of suspension.

### Growth under greenhouse conditions

Plants were grown for 6 weeks in a phytotron equipped with white and yellow light (photoperiod of 11 h of light, light intensity 505 μmol.m^–2.^s^–1^, stable room temperature 25 ± 3°C). Irrigation was performed every 48 h by capillarity with sterile tap water, with an experimental volume of 350 ml/tray.

### Evaluation of the effects of mercury on plant development

#### Plant biometry

For biometric parameter determination, after 6-week experiment, whole plants were harvested. Roots and shoots were washed with distilled water. With the recently harvested plants, the following parameters were measured: total weight (g), shoot weight (g), root weight (g), shoot length (cm), root length (cm), total number of leaves (N_*o*_), and total number of secondary roots (N_*o*_).

#### Plant antioxidant response

Enzymes were extracted at 4°C from 1 g of fresh sample, with a mortar and using 50 mg of polyvinylpolypyrrolidone (PVPP) and 10 ml of the following medium: 50 mm of K-phosphate buffer (pH 7.8) with 0.1 mm EDTA (for SOD, CAT, and APX). The same medium, supplemented with 10 mm of β-mercaptoethanol was used for GR. Superoxide dismutase (SOD) enzyme was measured based on SOD’S ability to inhibit the reduction of tetrazoyl nitro-blue (NBT) by photochemically generated superoxide radicals. A unit of turf is defined as the amount of enzyme needed to inhibit the rate of NBT reduction by 50% at 25°C (Burd et al., 2000). Catalase production was quantified using Aebi method (Aebi, 1984). H_2_O_2_ consumption was monitored for 1 min at 240 nm. This was carried out by mixing 50 mm of potassium phosphate buffer with 10 mm of H_2_O_2_ and 100 μl of the extract. APX content was measured in a 1 ml reaction containing 80 nm of potassium phosphate buffer, 2.5 mm of H_2_O_2_ and 1M sodium ascorbate. H_2_O_2_ was added to begin the reaction and absorbance reduction was measured for 1 min at 290 nm, to determine the oxidation ratio of ascorbate (Amako et al., 1994). Glutathione reductase (GR) enzyme was estimated spectrophotometrically, according to the Carlberg and Mannervik (1985) method at 25°C and 340 nm. To do this, the reaction mixture contained 50 mm of Tris-MgCl_2_ buffer, 3 mm, 1 mm of GSSG, 50 μl of enzyme, and 0.3 mm NADPH, added to initiate the reaction. Enzyme concentration was calculated with the initial rate of the reaction and the molar extinction coefficient of NADPH (ε340 = 6.22 mm^–1^ cm^–1^).

#### Mercury accumulation in plant

The root and shoot fraction of each replica were dried in dry heat furnaces at 60°C for 24 h. Each fraction was crushed and digested separately in acid medium (HNO_3_/HCl 2%/0.5% weight/volume) under pressure for the determination of trace elements according to the UNE-EN 13805 standard. The product of the digestion was then analyzed by inductively coupled plasma mass spectrometry (ICP-MS). Using a calibration curve, a correlation was established between the concentration of the standard (μg l^–1^) and the signal (ICP-MS) of mercury element. The element signal value in the 12 samples was interpolated on the calibration curve, resulting in the total concentration of the mercury in the sample. The mercury standard values for establishing the calibration line were as follows, expressed in μg.l^–1^: 0.00; 0.05; 0.10; 0.50; 1.00; 5.00; 10.00. Final units’ mg kg^–1^.


$$\text{Cf}\,\left(\frac{\mu \,\text{g}}{\text{Kg}}\right)=\text{X}\,\left(\frac{\mu \,\text{g}}{\text{L}}\right)\cdot \text{D}\cdot \frac{\text{V}\,\left(\text{mL}\right)}{\text{W}\,\left(\text{g}\right)}\cdot 10^{-3}$$

Cf (mg kg^–1^) is the metal content of the sample, X (μg l^–1^) corresponds to the interpolated experimental value or the extrapolated experimental value of the standard addition; D is the dilution factor; V (mL) is the volume of the flask, and W(g) to the weight of the sample.

### SAICEUPSM^T^ strain identification

#### Transmission electron microscopy (TEM)

To determine the size and shape of the analyzed strain, the Prism E scanning electron microscope (SEM) (Thermo Fisher Scientific Inc., Waltham, MA, USA) was used. The culture was observed in suspension. A drop of Formvar was used in transmission electron microscopy (TEM) Cu grids of mesh 200 as a sample support in the grid for TEM microscopy. The measurement conditions were working distance of 10 mm; 24 pA electron current; electron acceleration of 30 kV and size of 2 points, pressure of 375 Pa, at 4°C and a humidity of 50%. The electron microscopy images were obtained by the research support service (SAI) of “X-ray diffraction and scanning electron microscopy” (SAI-DRX-MEB) of the San Pablo CEU University (Madrid, Spain).

#### Biochemical tests

Oxi/Ferm Pluri Test^®^ (Liofilchem, Italy) was used. Next, the automatic characterization was carried out with the VITEK^®^ 2 equipment and with the VITEK^®^2 GN identification cards (bioMérieux, Marcy-l’Étoile, France). The motility of the bacterium was tested in Motility Test Agar, (Liofilchem, Italy). Antimicrobial sensitivity was determined using *E*-test in Müller Hinton agar (Pronadisa^®^, Madrid, Spain) using the following antibiotics: piperacillin and piperacillin with tazobactam, cefepime (bioMérieux, Marcy-l’Étoile, France); ceftazidime, imipenem, imipenem with EDTA, amikacin, gentamicin, and ciprofloxacin (Liofilchem, Italy).

#### Fatty acids

The analysis of cellular fatty acids was carried out in the Spanish Collection of Type Crops (CECT) at the University of Valencia, Spain. Cells were grown in M2 medium for 48 h, 30°C; extractions and determinations were carried out according to the standard protocol of the MIDI Microbial Identification System (Sasser, 1990) using a chromatograph Agilent 6850 (Agilent Technologies) following the method TSBA6 (MIDI, 2008, version 6.1. Newark, DE: MIDI Inc.).

#### Phylogenetic analysis

Matrix-assisted laser desorption/ionization–time of flight (MALDI-TOF) was used. It was carried out in the Vitek MS IND system (BioMérieux, Marcy-l’Étoile, France) at the Carlos III Health Institute (Majadahonda, Madrid). Slides were inoculated with a sterile handle. A total of 1 μl of the matrix solution (VITEK MS-CHCA: mixture of 3.10 g of 2.5-dihydroxy 36 benzoic acid dissolved in 100 ml of water-ethanol-acetonitrile in 1/1/1 ratio) was added to each well and allowed to dry at room temperature. the temperature. Mass spectra were generated with the Axima Assurance system (Shimadzu Corporation, Kyoto, Japan), using the Shimadzu Launchpad software program and the SARAMIS MS-ID v1 database application (AnagnosTee GmbH) for automatic measurement and identification. All strains were analyzed in duplicate. No pre-treatment was used before inoculation on the slide. High confidence identification was considered when the assessment was equal to or greater than 97%.

SAICEUPSM^T^ genomic DNA was extracted from fresh cells. Amplification of the *16S rRNA* gene was done and BLAST algorithm was used to search for similar sequences. An *in silico* genome analysis was carried out among the most closely related species, for this purpose the Type (Strain) Genome Server (TYGS) service was used using blast+ software. The resulting intergenic distances were used to infer a balanced evolution with branch support through FASTME 2.1.6.1. Compatibility was inferred from 100 pseudo-bootstrap replicas. Trees were rooted in the midpoint and visualized with PhyD3. For the calculation of ANI and AAI the tools available in JSpeciesWS and the ANI calculator^1^ were used.

#### Genome sequencing and bioinformatics analysis

For the extraction of genomic DNA, the QIAamp DNA Kit (QIAGEN^®^, Hilden, Germany) was used. SAICEUPSM^T^ genome was obtained by whole genome sequencing using an Illumina Miseq platform. A total of 200 ng of genomic DNA were used, measured by fluorimetry (Quant-iT Picogreen, Thermo Fisher). To make libraries, the NEB Next ultra II FS DNA preparation kit was used, according to the manufacturer’s protocol (New England Biolabs). The initial fragmentation time was 7.5 min, and the final PCR amplification was done with six cycles. The resulting DNA fragments were evaluated and quantified by bioanalyzer using a 7500 DNA (Agilent) chip. The libraries were quantified by qPCR using the master mix “Kapa-SYBR FAST qPCR kit for LightCycler480.” Libraries were sequenced in Illumina’s Miseq equipment following manufacturer’s instructions, in a pair end 2 × 300 type race using “Miseq reagent kit v3 600 cycles.”

The quality of the sequenced reads was processed using FastQC v.0.11.3 (Babraham Bioinformatics) (Andrews, 2010). They were filtered for low quality reads using Prinseq (Schmieder and Edwards, 2011) and adapter regions using CUTADAPT (Martin, 2011). To eliminate duplicate reads or those that were only present in forward or reverse, FASTQCollapser and FASTQIntersect were used. The *de novo* assembly of the genome was performed with SPAdes v.3.13 (Center for Algorithmic Biotechnology) (Bankevich et al., 2012), the metrics of the assemblies were obtained through SeqEditor (Hafez et al., 2021) and annotated using the Prokka software, version 1.13^2^ (Seemann, 2014) and Rapid using Subsystem Technology (RAST) version 2.0^3^ with predetermined parameters (Aziz et al., 2012). The genomes of the related strains were obtained from the Genbank database.

Additional bioinformatic analyses were performed. tRNAscan-SE v.2.0^4^ was used to predict tRNAs. Several clinically important antimicrobial resistance genes and virulence determinants were searched through functional annotation data generated from Rast, Prokka, and ResFinder 4.1 annotation lines. Several mercury resistance and plant growth promotion genes were searched through functional annotation data generated from Rast and Prokka annotation lines. KEGG software was used to examine the metabolic pathways of the strain, and PathogenFinder v.1.1., Rast and Prokka software were used to estimate pathogenicity.

### Statistical analysis

The Kolmogorov–Smirnov test was performed to check the normality of all variables. After that, for the analysis of the biometric data, an ANOVA test was carried out to analyze the plant response in the presence of SAICEUPSM^T^ in substrates under high and low mercury concentration. For those parameters that showed statistical significance (*p*-value ≤ 0.05), the multiple comparison test least significant difference (LSD) was performed, to identify if SAICEUPSM^T^ produced any significant variation in plant biometry, compared to controls. For the analysis of the phytoprotective capacity, a Kruskal–Wallis analysis was performed. For the evaluation of mercury accumulation in plants, the normality of the sample data was verified using the Shapiro–Wilk test. An ANOVA analysis was then performed to determine the existence of significant differences (*p*-value ≤ 0.05) between treatments. All statistical differences refer to the comparison of the variables when the plant has been inoculated, compared to their respective non-inoculated controls. SPSS v.27.0 software was used (Version 27.0 IBM Corp., Armonk, NY, USA).

## Data availability statement

The data presented in this study are deposited in the Genbank repository, accession number: JAMSHA000000000.

## Author contributions

PJ, VF, and MR: conceptualization and methodology. VF: software. PJ and MR: validation and supervision. DG, VF, and LG: formal analysis. DG, VF, LG, and MR: investigation. VF and LG: resources. PJ, VF, and LG: data curation. LG and MR: writing—original draft preparation and visualization. PJ, AP, and MR: writing—review and editing. AP and PJ: project administration and funding acquisition. All authors read and agreed to the published version of the manuscript.

## Footnotes

1 http://enve-omics.ce.gatech.edu/ani/, accessed 5 July 2022.

2
https://github.com/tseemann/prokka

3
https://rast.nmpdr.org/

4
http://lowelab.ucsc.edu/tRNAscan-SE/
