# Supplementary material for: Oxidative stress protection and growth promotion activity of Pseudomonas mercuritolerans sp. nov., in forage plants under mercury abiotic stress conditions
Source: Front Microbiol. 2022 Dec 6;13:1032901. doi: 10.3389/fmicb.2022.1032901 (PMC9763275; doi:10.3389/fmicb.2022.1032901)
Supplement: Supplementary file 1 [file Data_Sheet_1.docx]

**SUPPLEMENTARY MATERIAL**

**
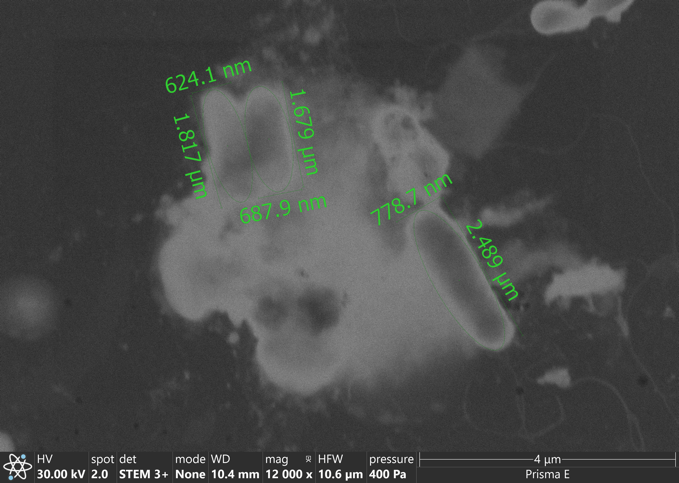
**

Figure S1 SAICEUPSMT transmission electron microscope images.

| 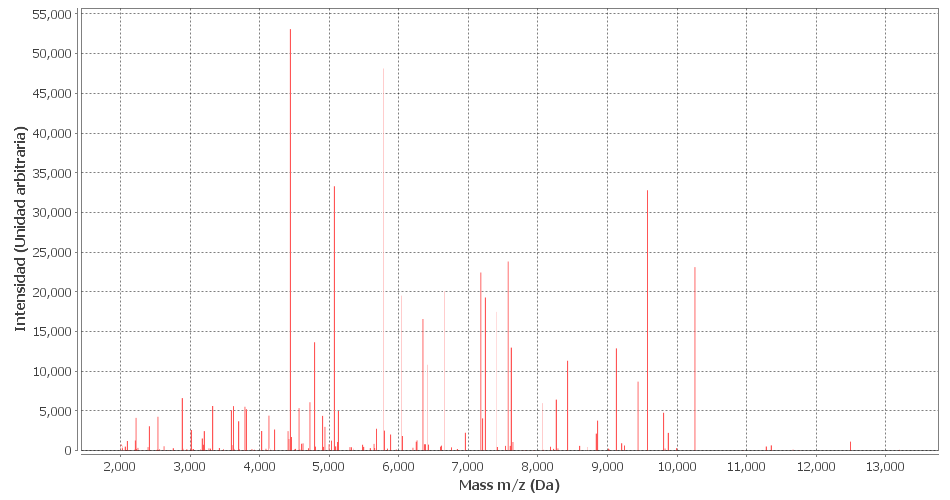 | M/Z  4,434  5,764  5,067  9,567  7,565  10,249  7,173  6,640  6,023  7,237 | INTENSITY  53,095.72  48,137.45  33,305.20  32,787.62  23,816.21  23,106.39  22,433.07  20,082.58  19,523.70  19,290.17 |
| --- | --- | --- |
| **Figure S2.** Mass spectrum of strain SAICEUPSM^T^ obtained by using the VITEK MS automated mass spectrometry system (bioMérieux). The horizontal axis shows mass/charge ratio (Da) and the vertical axis shows the relative intensities of ions (%). Peaks >12,500 Da are not shown due to their low intensity. | | |

Figure S2

**Table S1: ANIb**

| **#ANIb and aligned percentage** |  |  |  |  |  |  |  |  |  |  |  |  |  |
| --- | --- | --- | --- | --- | --- | --- | --- | --- | --- | --- | --- | --- | --- |
|  | 1 | 2 | 3 | 4 | 5 | 6 | 7 | 8 | 9 | 10 | 11 | 12 | 13 |
| ***Pseudomonas atacamensis* M7D1 [T]** | * | 95.72 [85.51] | 95.67 [84.37] | 95.13 [82.19] | 91.61 [82.97] | 88.43 [78.63] | 87.82 [79.99] | 86.95 [78.84] | 86.78 [78.45] | 86.61 [77.65] | 86.52 [78.81] | 86.20 [74.60] | 94.65 [85.68] |
| ***Pseudomonas_iranensis*_SWRI54** | 95.72 [87.32] | * | 95.07 [85.37] | 94.67 [82.75] | 91.59 [84.06] | 88.53 [79.11] | 88.02 [81.05] | 86.99 [80.27] | 86.84 [80.01] | 86.61 [79.27] | 86.72 [79.55] | 86.22 [76.19] | 94.83 [87.30] |
| ***Pseudomonas_triticicola*_SWRI88** | 95.83 [89.72] | 95.25 [88.76] | * | 95.05 [83.54] | 91.79 [85.72] | 88.64 [80.72] | 87.79 [83.16] | 86.82 [82.12] | 86.83 [81.22] | 86.59 [80.86] | 86.63 [81.32] | 86.25 [76.46] | 94.68 [88.38] |
| ***Pseudomonas_siliginis*_SWRI31** | 95.56 [88.62] | 95.08 [87.25] | 95.20 [84.81] | * | 91.67 [83.93] | 88.64 [79.83] | 87.98 [82.30] | 86.98 [80.46] | 86.87 [80.74] | 86.63 [80.17] | 86.79 [80.04] | 86.13 [77.66] | 94.41 [86.71] |
| ***Pseudomonas_moraviensis*_BS3668** | 91.62 [84.34] | 91.59 [83.79] | 91.70 [82.16] | 91.37 [79.09] | * | 88.31 [79.05] | 87.93 [80.76] | 86.84 [81.12] | 86.59 [80.98] | 86.55 [80.22] | 86.78 [81.41] | 86.07 [75.95] | 91.63 [84.45] |
| ***Pseudomonas_khorasanensis*_SWRI153** | 88.72 [85.38] | 88.70 [83.79] | 88.71 [83.03] | 88.56 [80.49] | 88.41 [84.63] | * | 87.51 [83.91] | 86.38 [82.10] | 86.46 [81.38] | 86.31 [81.39] | 86.50 [81.74] | 86.09 [79.28] | 88.74 [84.66] |
| ***Pseudomonas_hamedanensis*_SWRI65** | 87.74 [81.94] | 87.96 [81.27] | 87.60 [80.05] | 87.62 [78.19] | 87.86 [81.27] | 87.24 [79.45] | * | 86.67 [81.71] | 86.67 [81.03] | 86.56 [80.58] | 86.45 [80.43] | 86.10 [77.02] | 87.83 [81.40] |
| ***Pseudomonas_granadensis*_LMG_27940** | 86.94 [82.29] | 87.02 [82.02] | 86.68 [80.93] | 86.66 [78.23] | 86.87 [83.17] | 86.18 [79.16] | 86.73 [83.18] | * | 87.21 [83.74] | 87.01 [82.98] | 87.08 [82.59] | 86.34 [76.86] | 86.88 [82.61] |
| ***Pseudomonas_tensinigenes*_ZA_5.3** | 86.48 [74.84] | 86.55 [74.68] | 86.45 [72.85] | 86.35 [71.56] | 86.25 [75.61] | 86.04 [71.61] | 86.38 [75.38] | 86.87 [76.20] | * | 93.34 [88.21] | 90.80 [83.23] | 87.74 [76.19] | 86.36 [75.81] |
| ***Pseudomonas_crudilactis*_UCMA_17988** | 86.21 [74.05] | 86.21 [73.74] | 86.02 [72.44] | 86.03 [70.77] | 86.02 [74.94] | 85.74 [71.09] | 86.16 [74.50] | 86.68 [75.13] | 93.28 [87.96] | * | 90.80 [82.89] | 87.59 [75.68] | 86.08 [74.64] |
| ***Pseudomonas_neuropática*_P155** | 86.36 [75.94] | 86.53 [74.79] | 86.34 [73.74] | 86.29 [71.50] | 86.50 [76.97] | 86.17 [72.05] | 86.29 [75.24] | 86.88 [76.07] | 90.95 [84.25] | 90.98 [84.46] | * | 87.84 [76.62] | 86.36 [76.23] |
| ***Pseudomonas_*baetica_LMG_25716** | 86.03 [68.27] | 86.01 [67.47] | 85.87 [65.42] | 85.85 [65.67] | 85.84 [68.17] | 85.85 [66.43] | 85.97 [68.06] | 86.21 [67.01] | 87.82 [72.86] | 87.79 [72.39] | 87.97 [72.26] | * | 85.95 [68.84] |
| **SAICEU98^T^** | 94.56 [84.40] | 94.67 [84.22] | 94.29 [82.20] | 93.98 [79.29] | 91.41 [81.81] | 88.40 [76.62] | 87.79 [78.42] | 86.79 [77.73] | 86.55 [78.35] | 86.46 [77.31] | 86.42 [77.92] | 86.17 [74.33] | * |

*Pseudomonas atacamensis* M7D1 [T] (1), *Pseudomonas_iranensis*_SWRI54 (2), *Pseudomonas_triticicola*_SWRI88 (3), *Pseudomonas_siliginis*_SWRI31 (4), *Pseudomonas_moraviensis*_BS3668 (5), *Pseudomonas_khorasanensis*_SWRI153 (6), *Pseudomonas_hamedanensis*_SWRI65 (7), *Pseudomonas_granadensis*_LMG_27940 (8), *Pseudomonas_tensinigenes*_ZA_5.3 (9), *Pseudomonas_crudilactis*_UCMA_17988 (10), *Pseudomonas_neuropática*_P155 (11), *Pseudomonas_baetica*_LMG_25716 (12), SAICEUPSM^T^ (13)

**Table S2. TETRA results**

|  | 1 | 2 | 3 | 4 | 5 | 6 | 7 | 8 | 9 | 10 | 11 | 12 | **13** |
| --- | --- | --- | --- | --- | --- | --- | --- | --- | --- | --- | --- | --- | --- |
| ***Pseudomonas atacamensis* M7D1 [T]** | * | 0.99961 | 0.99927 | 0.99896 | 0.99654 | 0.9943 | 0.99246 | 0.99404 | 0.99049 | 0.99042 | 0.98905 | 0.98854 | **0.99696** |
| ***Pseudomonas_iranensis*_SWRI54** | 0.99961 | * | 0.99945 | 0.99892 | 0.99706 | 0.9939 | 0.99195 | 0.99394 | 0.99125 | 0.99104 | 0.98991 | 0.98877 | **0.99752** |
| ***Pseudomonas_triticicola*_SWRI88** | 0.99927 | 0.99945 | * | 0.99892 | 0.99697 | 0.99393 | 0.99199 | 0.99412 | 0.99101 | 0.99098 | 0.98926 | 0.98843 | **0.99702** |
| ***Pseudomonas_siliginis*_SWRI31** | 0.99896 | 0.99892 | 0.99892 | * | 0.99604 | 0.99502 | 0.99279 | 0.99251 | 0.98956 | 0.98954 | 0.98776 | 0.98904 | **0.99687** |
| ***Pseudomonas_moraviensis*_BS3668** | 0.99654 | 0.99706 | 0.99697 | 0.99604 | * | 0.99299 | 0.99358 | 0.99582 | 0.99016 | 0.99047 | 0.99159 | 0.98816 | **0.99553** |
| ***Pseudomonas_khorasanensis*_SWRI153** | 0.9943 | 0.9939 | 0.99393 | 0.99502 | 0.99299 | * | 0.99566 | 0.99184 | 0.98716 | 0.9874 | 0.98711 | 0.99153 | **0.9916** |
| ***Pseudomonas_hamedanensis*_SWRI65** | 0.99246 | 0.99195 | 0.99199 | 0.99279 | 0.99358 | 0.99566 | * | 0.99445 | 0.98545 | 0.98712 | 0.98733 | 0.99025 | **0.98999** |
| ***Pseudomonas_granadensis*_LMG_27940** | 0.99404 | 0.99394 | 0.99412 | 0.99251 | 0.99582 | 0.99184 | 0.99445 | * | 0.9889 | 0.99014 | 0.99077 | 0.98734 | **0.99061** |
| ***Pseudomonas_tensinigenes*_ZA_5.3** | 0.99049 | 0.99125 | 0.99101 | 0.98956 | 0.99016 | 0.98716 | 0.98545 | 0.9889 | * | 0.99914 | 0.99787 | 0.99191 | **0.98773** |
| ***Pseudomonas_crudilactis*_UCMA_17988** | 0.99042 | 0.99104 | 0.99098 | 0.98954 | 0.99047 | 0.9874 | 0.98712 | 0.99014 | 0.99914 | * | 0.99839 | 0.99107 | **0.98713** |
| ***Pseudomonas_neuropática*_P155** | 0.98905 | 0.98991 | 0.98926 | 0.98776 | 0.99159 | 0.98711 | 0.98733 | 0.99077 | 0.99787 | 0.99839 | * | 0.99119 | **0.98652** |
| ***Pseudomonas_baetica*_LMG_25716** | 0.98854 | 0.98877 | 0.98843 | 0.98904 | 0.98816 | 0.99153 | 0.99025 | 0.98734 | 0.99191 | 0.99107 | 0.99119 | * | **0.98784** |
| **SAICEU98^T^** | 0.99696 | 0.99752 | 0.99702 | 0.99687 | 0.99553 | 0.9916 | 0.98999 | 0.99061 | 0.98773 | 0.98713 | 0.98652 | 0.98784 | ***** |

*Pseudomonas atacamensis* M7D1 [T] (1), *Pseudomonas_iranensis*_SWRI54 (2), *Pseudomonas_triticicola*_SWRI88 (3), *Pseudomonas_siliginis*_SWRI31 (4), *Pseudomonas_moraviensis*_BS3668 (5), *Pseudomonas_khorasanensis*_SWRI153 (6), *Pseudomonas_hamedanensis*_SWRI65 (7), *Pseudomonas_granadensis*_LMG_27940 (8), *Pseudomonas_tensinigenes*_ZA_5.3 (9), *Pseudomonas_crudilactis*_UCMA_17988 (10), *Pseudomonas_neuropática*_P155 (11), *Pseudomonas_baetica*_LMG_25716 (12), SAICEUPSM^T^ (13)

**Table S3. Intergenic distance**

|  | 1 | 2 | 3 | 4 | 5 | 6 | 7 | 8 | **9** | 10 | 11 | 12 | 13 |
| --- | --- | --- | --- | --- | --- | --- | --- | --- | --- | --- | --- | --- | --- |
| ***Pseudomonas_tensinigenes_ZA_5.3*** | * | 0.06693 | 0.08470 | 0.11775 | 0.12829 | 10.012.032 | 10.012.380 | 10.012.248 | **10.012.356** | 10.012.286 | 10.012.370 | 10.012.762 | 10.012.381 |
| ***Pseudomonas_crudilactis_UCMA_17988*** | 0.06693 | * | 0.08593 | 0.11899 | 0.12953 | 10.012.156 | 10.012.504 | 10.012.372 | **10.012.480** | 10.012.409 | 10.012.494 | 10.012.885 | 10.012.05 |
| ***Pseudomonas_neuropática_P155*** | 0.08470 | 0.08593 | * | 0.11856 | 0.12909 | 10.012.112 | 10.012.460 | 10.012.329 | **10.012.437** | 10.012.366 | 10.012.451 | 10.012.842 | 10.012.462 |
| ***Pseudomonas_baetica_LMG_25716*** | 0.11775 | 0.11899 | 0.11856 | * | 0.13202 | 10.012.405 | 10.012.753 | 10.012.621 | **10.012.729** | 10.012.659 | 10.012.743 | 10.013.135 | 10.012.755 |
| ***Pseudomonas_granadensis_LMG_27940*** | 0.12829 | 0.12953 | 0.12909 | 0.13202 | * | 10.011.911 | 10.012.260 | 10.012.128 | **10.012.236** | 10.012.165 | 10.012.250 | 10.012.641 | 10.012.261 |
| ***Pseudomonas atacamensis M7D1 [T]*** | 10.012.032 | 10.012.156 | 10.012.112 | 10.012.405 | 10.012.911 | * | 0.03939 | 0.04090 | **0.04338** | 0.04849 | 0.07897 | 0.11087 | 0.11464 |
| ***Pseudomonas_iranensis_SWRI54*** | 10.012.380 | 10.012.504 | 10.012.460 | 10.012.753 | 10.012.260 | 10.012.939 | * | 0.04438 | **0.04686** | 0.05197 | 0.08244 | 0.11435 | 0.11812 |
| ***Pseudomonas_triticicola_SWRI88*** | 10.012.248 | 10.012.372 | 10.012.329 | 10.012.621 | 10.012.128 | 0.04090 | 0.04438 | * | **0.04554** | 0.05065 | 0.08113 | 0.11303 | 0.11681 |
| ***Pseudomonas_siliginis_SWRI31*** | 10.012.356 | 10.012.480 | 10.012.437 | 10.012.729 | 10.012.236 | 0.04338 | 0.04686 | 0.04554 | ***** | 0.05173 | 0.08221 | 0.1111411 | 0.11789 |
| ***SAICEUPSMT*** | 10.012.286 | 10.012.409 | 10.012.366 | 10.012.659 | 10.012.165 | 0.04849 | 0.05197 | 0.05065 | **0.05173** | * | 0.08150 | 0.11341 | 0.11718 |
| ***Pseudomonas_moraviensis_BS3668*** | 10.012.379 | 10.012.494 | 10.012.451 | 10.012.743 | 10.012.250 | 0.07896 | 0.08244 | 0.08113 | **0.08221** | 0.08150 | * | 0.011425 | 0.11803 |
| ***Pseudomonas_khorasanensis_SWRI153*** | 10.012.762 | 10.012.885 | 10.012.842 | 10.012.135 | 10.012.641 | 0.11087 | 0.11435 | 0.11303 | **0.11411** | 0.11341 | 0.11425 | * | 0.12194 |
| ***Pseudomonas_hamedanensis_SWRI65*** | 10.012.381 | 10.012.505 | 10.012.462 | 10.012.755 | 10.012.261 | 10.011.464 | 10.011.812 | 10.011.681 | **10.011.789** | 10.011.718 | 10.011.803 | 10.011.194 | * |

*Pseudomonas tensinigenes* ZA_5.3 (1), *Pseudomonas crudilactis* UCMA_17988 (2), *Pseudomonas_neuropática* P155 (3), *Pseudomonas baetica* LMG_25716 (4), *Pseudomonas granadensis* LMG_27940 (5), *Pseudomonas atacamensis* M7D1 [T] (6), *Pseudomonas iranensis* SWRI54 (7), *Pseudomonas triticicola* SWRI88 (8), *Pseudomonas siliginis* SWRI31 (9), SAICEUPSM^T^ (10), *Pseudomonas moraviensis* BS3668 (11), *Pseudomonas khorasanensis* SWRI153 (12), *Pseudomonas hamedanensis* SWRI65 (13

**Table S4.** Minimum inhibitory concentration (MIC) for the different antibiotics

| Antibiotic | SAICEUPSMT MIC ($\mu$g.mL^-1^) |
| --- | --- |
| Piperacillin | ≥128 (R) |
| Piperacillin/Tazobactam | 32 (R) |
| Ceftazidime | 4 (S) |
| Cefepime | 4 (S) |
| Aztreonam | ≥64 (R) |
| Doripenem | 2 (I) |
| Imipenem | 2 (S) |
| Meropenem | 1 (S) |
| Gentamycin | ≤1 (S) |
| Tobramycin | ≤1 (S) |
| Ciprofloxacin | ≤10.25 (S) |
| Levofloxacin | ≤10,12 (S) |
| Colistin | 2 (S) |
| Fosfomycin | 128 (R) |

**Table S5:** SAICEUPSM^T^ genome annotation information using RAST server.

| Categories | Subcategories | SAICEUPSM^T^ | M7D1 |
| --- | --- | --- | --- |
| Cofactors, Vitamis, Prosthetic Groups, Pigments | Biotin | 15 | 17 |
|  | Thiamin biosynthesis | 9 | 8 |
|  | Tetrapyrroles | 41 | 39 |
|  | Riboflavin, FMN, FAD | 37 | 32 |
|  | Pyridoxine | 15 | 14 |
|  | NAD and NADP | 15 | 16 |
|  | Folate and pterines | 59 | 60 |
|  | Lipoic acid | 2 | 3 |
|  | Coenzyme A | 13 | 12 |
| Cell Wall and Capsule | Capsular and extracellular polysaccharides | 31 | 27 |
|  | Gram-Negative cell wall components | 6 | 6 |
|  | Cell Wall and Capsule | 16 | 16 |
| Virulencia, Enfermedad y Defensa | Bacteriocinas, peptidos antibacterianos sintetizados ribosomalmente | 2 | 2 |
|  | Resistencia a antibioticos y compuestos tóxicos | 52 | 44 |
|  | Invasión y resistencia intracelular | 14 | 13 |
| Potassium homeostasis |  | 11 | 13 |
| Miscellaneous | Plant-Prokaryote DOE project | 17 | 17 |
|  | Phosphoglycerate mutase protein family | 3 | 3 |
|  | DedA family of inner membrane proteins | 2 | 1 |
|  | Muconate lactonizing enzyme family | 1 | 1 |
|  | Bacillus subtilis scratch - gjo | 2 | 2 |
|  | Broadly distributed proteins not in subsystems | 6 | 6 |
| Phages, Prophages | Phage capsid proteins | 1 | 0 |
|  | Phage lysis modules | 0 | 2 |
|  | Phage DNA synthesis | 1 | 1 |
| Membrane Transport | Protein secretion system, Type II | 14 | 14 |
|  | ABC transporters | 4 | 4 |
|  | Protein translocation across cytoplasmic membrane | 4 | 4 |
|  | Protein secretion system, Type V | 2 | 3 |
|  | Protein secretion system, Type I | 12 | 11 |
|  | Cation transporters | 18 | 24 |
|  | Uni-Sym-and Antiporters | 9 | 9 |
|  | Membrane Transport – no subcategory | 22 | 18 |
|  | TRAP transporters | 9 | 9 |
|  | Protein and nucleoprotein secretion system, Type IV | 24 | 24 |
| Iron acquisition and metabolism | Siderophores | 15 | 14 |
|  | Iron acquisition and metabolism – no subcategory | 11 | 11 |
| RNA Metabolism | RNA processing and modification | 31 | 34 |
|  | Transcription | 26 | 25 |
|  | RNA Metabolism | 1 | 1 |
| Nucleosides and Nucleotides | Pyrimidines | 23 | 23 |
|  | Purines | 59 | 64 |
|  | Nucleosides and nucleotides – no subcategory | 9 | 15 |
|  | Detoxification | 6 | 6 |
| Protein Metabolism | Protein folding | 25 | 24 |
|  | Selenoproteins | 5 | 8 |
|  | Protein biosynthesis | 151 | 154 |
|  | Protein processing and modification | 12 | 12 |
|  | Protein degradation | 27 | 27 |
| Motility and Chemotaxis | Flagelar motility in Prokaryota | 68 | 69 |
| Regulation and Cell signaling | Regulation and Cell signaling – no subcategory | 42 | 46 |
|  | Quorum sensing and biofilm formation | 4 | 9 |
|  | Programmed Cell Death and Toxin-antitoxin Systems | 7 | 10 |
| Secondary Metabolism | Plant Hormones | 4 | 5 |
| DNA Metabolism | DNA repair | 75 | 73 |
|  | DNA Metabolism – no subcategory | 14 | 11 |
|  | DNA replication | 0 | 9 |
|  | DNA uptake, competence | 3 | 3 |
| Fatty Acid, Lipids, and Isoprenoids | Phospholipids | 28 | 30 |
|  | Triacylglycerols | 3 | 3 |
|  | Fatty acids | 35 | 39 |
|  | Polyhydroxybutyrate metabolism | 24 | 29 |
|  | Isoprenoids | 11 | 11 |
| Nitrogen Metabolism |  | 14 | 13 |
| Dormancy and Sporulation |  | 2 | 2 |
|  | Biotin | 0 | 17 |
| Respiration | ATP synthases | 9 | 9 |
|  | Electron accepting | 9 | 0 |
|  | Electron accepting reactions | 38 | 37 |
|  | Electron donating reactions | 40 | 39 |
|  | Respiration | 45 | 42 |
| Stress Response | Osmotic stress | 13 | 13 |
|  | Oxidative stress | 57 | 60 |
|  | Detoxification | 12 | 11 |
|  | Stress Response | 20 | 27 |
|  | Periplasmic Stress | 6 | 6 |
| Metabolism of Aromatic Compounds | Peripheral pathways for catabolism of aromatic compounds | 27 | 31 |
|  | Metabolism of central aromatic intermediates | 32 | 33 |
|  | Metabolism of Aromatic Compounds | 6 | 5 |
| Amino Acids and Derivatives | Glutamine, glutamate, aspartate, asparagine; ammonia assimilation | 28 | 28 |
|  | Histidine, Metabolism | 25 | 16 |
|  | Arginine; urea cycle, polyamines | 104 | 101 |
|  | Lysine, threonine, methionine, and cysteine | 80 | 79 |
|  | Amino Acids and Derivatives | 2 | 2 |
|  | Branched-chain amino acids | 85 | 80 |
|  | Aromatic amino acids and Derivatives | 2 | 81 |
|  | Proline and 4-hydroxyproline | 15 | 15 |
|  | Alanine, serine, and glycine | 70 | 71 |
| Sulfur Metabolism | Sulfur Metabolism | 8 | 9 |
|  | Organic sulfur assimilation | 17 | 17 |
| Phosphorus Metabolism |  | 36 | 35 |
| Carbohydrates | Central carbohydrate metabolism | 98 | 90 |
|  | Aminosugars | 4 | 4 |
|  | Di – and oligosaccharides | 19 | 13 |
|  | One-carbon Metabolism | 6 | 6 |
|  | Organic acids | 29 | 40 |
|  | Fermentation | 38 | 41 |
|  | Sugar alcohols | 11 | 11 |
|  | Carbohydrates | 2 | 3 |
|  | Polysaccharides | 4 | 4 |
|  | Monosaccharides | 51 | 51 |

**Figure S3. A)** draft map of whole-genome and distribution of annotated genes of strain SAICEUPSM^T^ sp. **a** Genomic circle diagram of a new strain SAICEU98^T^. **B)** Annotate SAICEUPSM^T^ genome information using RAST server.

**A**

**
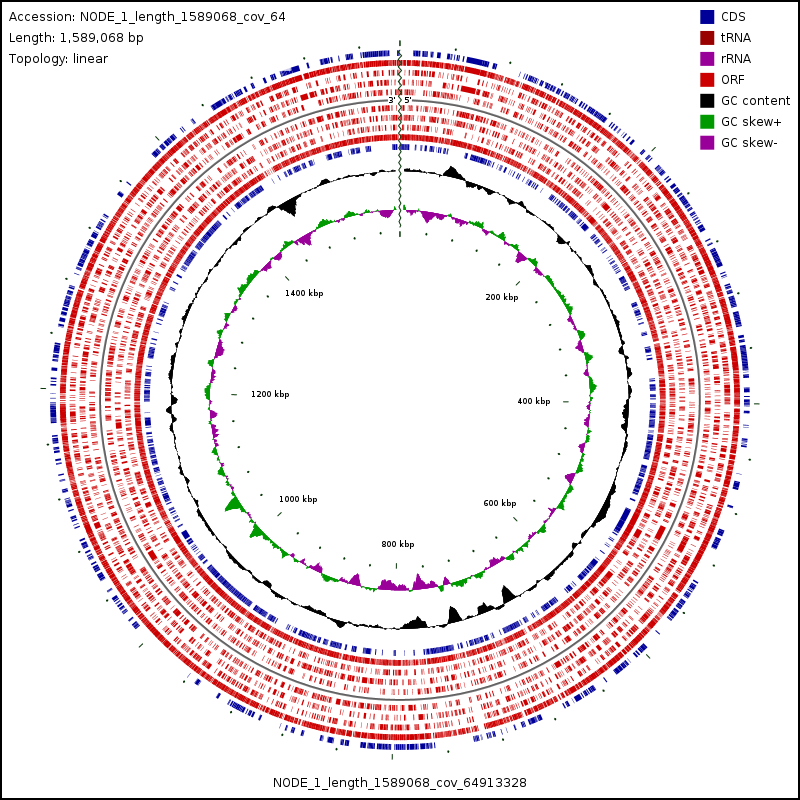
**

**B**

**
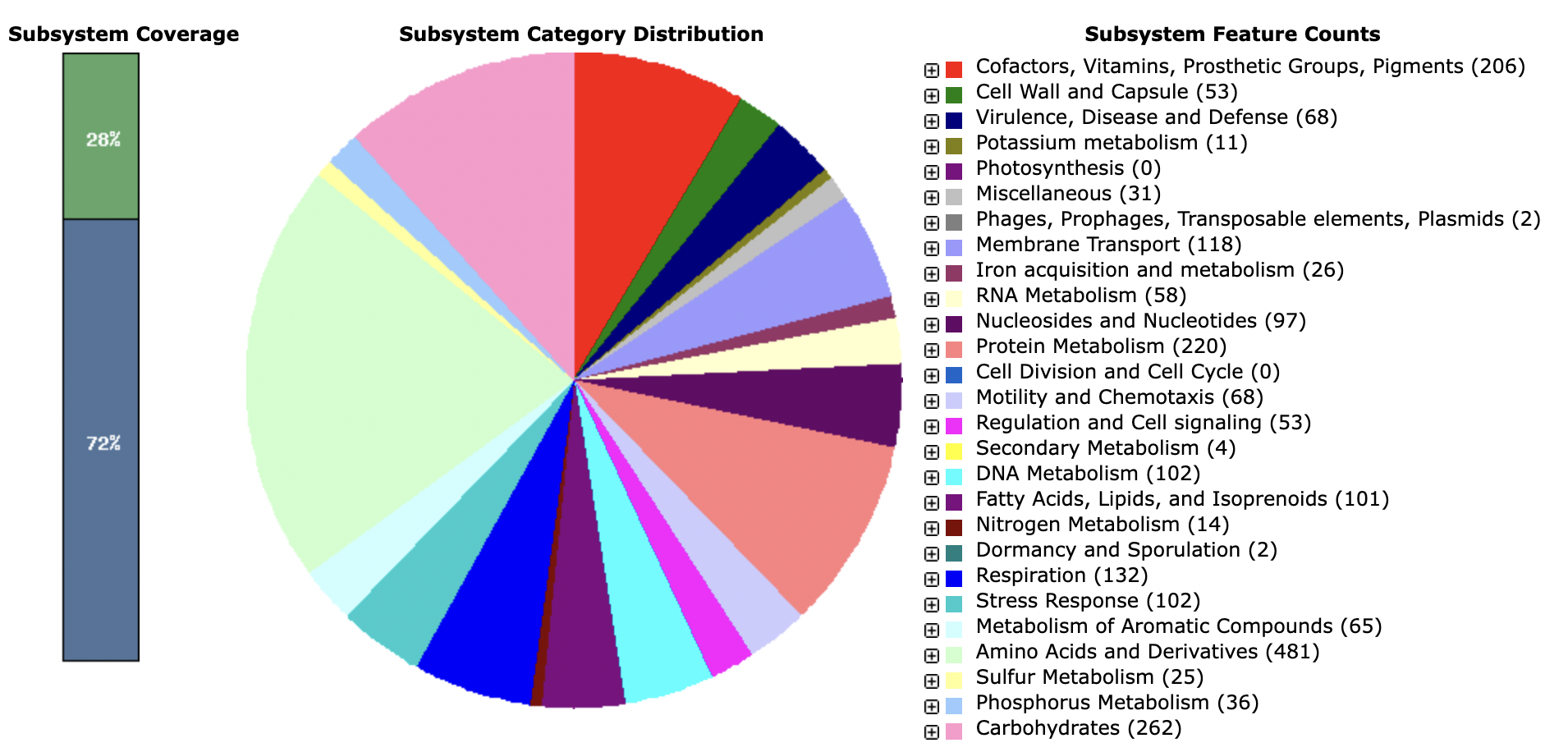
**

**Figure S4.** KEGG Mapper reconstruction result and *Mer operon* structure.

**A**

|  |
| --- |

|  | **Human Diseases** |
| --- | --- |
|  | **Organismal Systems** |
|  | **Cellular Processes** |
|  | **Environmental InformationProcessing** |
|  | **Genetic Information Processing** |
|  | **Metabolism** |

B


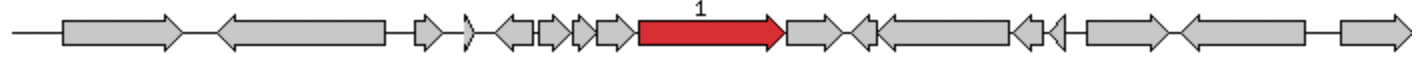


KEGG annotation class distribution. **A)** KEGG annotation statistics chart at Level 2. The horizontal axis is the number of genes, the vertical axis represents the name of the Level 2 pathway, and the number on the right side of the column is the number of genes annotated to the Level 2 pathway. **B)** Chromosomal region of the mercury operon.

**Table S6.** metabolic pathways KofamKOALA analysis.

| **Metabolic pathway** | **Number of genes** |
| --- | --- |
| **Metabolism** |  |
| Global and overview maps | 1796 |
| Carbohydrate metabolism | 310 |
| Energy metabolism | 158 |
| Lipid metabolism | 70 |
| Nucleotide metabolism | 94 |
| Amino acid metabolism | 304 |
| Metabolism of other amino acids | 78 |
| Glycan biosynthesis and metabolism | 70 |
| Metabolism of cofactors and vitamins | 187 |
| Metabolism of terpenoids and polyketides | 44 |
| Biosynthesis of other secondary metabolites | 54 |
| Xenobiotics biodegradation and metabolism | 74 |
| **Genetic Information Processing** |  |
| Transcription | 5 |
| Translation | 81 |
| Folding, sorting and degradation | 48 |
| Replication and repair | 79 |
| **Environmental Information Processing** |  |
| Membrane transport | 177 |
| Signal transduction | 154 |
| Signaling molecules and interaction | 1 |
| **Cellular Processes** |  |
| Transport and catabolism | 11 |
| Cell growth and death | 29 |
| Cellular community - prokaryotes | 140 |
| Cell motility | 59 |
| **Organismal Systems** |  |
| Immune system | 6 |
| Endocrine system | 16 |
| Circulatory system | 3 |
| Digestive system | 4 |
| Excretory system | 1 |
| Nervous system | 8 |
| Aging | 13 |
| Environmental adaptation | 13 |
| **Human Diseases** |  |
| Cancer: overview | 24 |
| Cancer: specific types | 6 |
| Infectious disease: viral | 11 |
| Infectious disease: bacterial | 29 |
| Infectious disease: parasitic | 4 |
| Immune disease | 1 |
| Neurodegenerative disease | 44 |
| Substance dependence | 3 |
| Cardiovascular disease | 17 |
| Endocrine and metabolic disease | 12 |
| Drug resistance: antimicrobial | 34 |
| Drug resistance: antineoplastic | 7 |

**Table S7.** Heavy metal resistance genes (Rapid annotation using Subsytem Technology)

| Subsystem | Gene | Functional Role |
| --- | --- | --- |
| Copper homeostasis  Copper tolerance | *CIA  *- CIA*  *- CSA* | 1. Lead, cadmium, zinc, and mercury transporting ATPase (EC 3.6.3.3) (EC 3.6.3.5); Copper-translocating P-type ATPase (EC 3.6.3.4). 2. Type cbb3 cytochrome oxidase biogenesis protein CcoI; Copper-translocating P-type ATPase (EC 3.6.3.4) |
|  | *clfA* | Multidrug resistance transporter, Bcr/CflA family |
|  | MO | Multicopper oxidase |
|  | CT | Copper tolerance protein |
|  | *HL  *- ccmF*  *- ccmH* | 1. Cytochrome c heme lyase subunit CcmF. 2. Cytochrome c heme lyase subunit CcmH |
|  | *copC* | Copper resistance protein CopC |
|  | *copD* | Copper resistance protein CopD |
|  | *copG* | CopG protein |
|  | *CRB* | Copper resistance protein B |
|  | *cusS* | Copper sensory histidine kinase CusS |
|  | *cusR* | Copper-sensing two-component system response regulator CusR |
|  | *corC* | Magnesium and cobalt efflux protein CorC |
|  | *cutE* | Apolipoprotein N-acyltransferase (EC 2.3.1.-) / Copper homeostasis protein CutE |
| Cobalt-zinc-cadmium resistance | **czcD*  *- czcR* | Cobalt-zinc-cadmium resistance protein |
|  | **czcR*  *- czcR*  *- cusR* | 1. Copper-sensing two-component system response regulator CusR 2. DNA-binding heavy metal response regulator |
|  | **HMHK*  *- HMHK*  *- cusS* | 1. Heavy metal sensor histidine kinase 2. Copper sensory histidine kinase CusS |
|  | **TR* | 1. Heavy metal sensor histidine kinase 2. Transcriptional regulator, MerR family 3. Heavy metal resistance transcriptional regulator HmrR |
| Resistance to chromium compounds |  | Chromate transport protein ChrA |

**Table S8.** Antibiotic resistance genes, using RAST server.

| **Streptothricin resistance** | | | | |
| --- | --- | --- | --- | --- |
| SAICEUPSMT | | | | |
| Function | Strand | Start | Stop | Size |
| Streptothricin acetyltransferase, *Streptomyces lavendulae* type | + | 377196 | 377810 | 615 |
| Ps. atacamensis M7D1 | | | | |
| Function | Strand | Start | Stop | Size |
| Streptothricin acetyltransferase, *Streptomyces lavendulae* type | + | 22841 | 23455 | 615 |
| **Resistance to fluoroquinolones** | | | | |
| SAICEUPSM^T^ | | | | |
| DNA gyrase subunit A (EC 5.99.1.3) | + | 172568 | 169914 | 2655 |
| DNA gyrase subunit B (EC 5.99.1.3) | + | 700393 | 702810 | 2418 |
|  | + | 3 | 455 | 453 |
| *Ps. atacamensis* M7D1 | | | | |
| DNA gyrase subunit A (EC 5.99.1.3) | + | 774594 | 777248 | 2655 |
| DNA gyrase subunit B (EC 5.99.1.3) | + | 653225 | 655432 | 2208 |
|  | + | 655422 | 655604 | 183 |
| **Fosfomycin resistance** | | | | |
| SAICEUPSM^T^ | | | | |
| Fosfomycin resistance protein FosA | + | 393355 | 393771 | 417 |
| *Ps. atacamensis* M7D1 | | | | |
| Fosfomycin resistance protein FosA | + | 690385 | 690801 | 417 |
| **Beta-lactamases** | | | | |
| SAICEUPSM^T^ | | | | |
| Metal-dependent hydrolases of the beta-lactamase superfamily I | + | 446256 | 447017 | 762 |
| *Ps. atacamensis* M7D1 | | | | |
| Metal-dependent hydrolases of the beta-lactamase superfamily I | + | 743514 | 744275 | 762 |
| **Multidrug Resistance Efflux Pumps** | | | | |
| SAICEUPSM^T^ | | | | |
| Multidrug and toxin extrusion (MATE) family efflux pump YdhE/NorM, homolog | + | 429479 | 430981 | 1503 |
| Multi antimicrobial extrusion protein (Na(+)/drug antiporter), MATE family of MDR efflux pumps | - | 10519 | 9110 | 1410 |
| Macrolide-specific efflux protein MacA | - | 163885 | 162722 | 1164 |
| RND multidrug efflux transporter; Acriflavin resistance protein | + | 3 | 617 | 615 |
|  | - | 533 | 41 | 513 |
|  | - | 422 | 3 | 420 |
| *Ps. atacamensis* M7D1 | | | | |
| Multidrug and toxin extrusion (MATE) family efflux pump YdhE/NorM, homolog | + | 431144 | 432646 | 1503 |
| Multi antimicrobial extrusion protein (Na(+)/drug antiporter), MATE family of MDR efflux pumps | + | 520929 | 522338 | 1410 |
| Macrolide-specific efflux protein MacA | - | 157757 | 156594 | 1164 |

**Table S9.** Virulence genes found in SAICEUPSM^T^ strain, directed search.

| Description of the activity | Function | Genetic annotation | Gene Names |
| --- | --- | --- | --- |
| Adherence | Mobility | Flagellin | (flgG) |
|  | Mobility | Flagellin | (*flgH*) |
|  | Mobility | Flagellin | (*flgB*) |
|  | Mobility | Flagellin | (*flgC*) |
|  | Mobility | Flagellin | (*flgI*) |
|  | Mobility | Flagellin | (*flgL*) |
|  | Mobility | Flagellin | (*flgA*) |
|  | Mobility | Flagellin | (*flhA*) |
|  | Mobility | Flagellin | (*flhB*) |
|  | Mobility | Flagellin | (*motC*) |
|  | Mobility | Flagellin | (*motA*) |
|  | Mobility | Flagellin | (*motD*) |
|  | Mobility | Flagellin | (*fliF*) |
|  | Mobility | Flagellin | (*fliN*) |
|  | Mobility | Flagellin | (*fliQ*) |
|  | Mobility | Flagellin | (*fliI*) |
|  | Mobility | Flagellin | (*fliH*) |
|  | Mobility | Flagellin | (*fliP*) |
|  | Mobility | Flagellin | (*fliL*) |
|  | Mobility | Flagellin | (*fliE*) |
|  | Mobility | Flagellin | (*fliA*) |
|  | Mobility | Flagellin | (*fliS*) |
|  | Mobility | Tipe IV biosíntesis de pili | (*pilB*) |
|  | Mobility | Tipe IV biosíntesis de pili | (*pilT*) |
| Antiphagocytosis | *Biofilm* | Alginate biosynthesis | (*algL*) |
|  | *Biofilm* | Alginate biosynthesis | (*algK*) |
|  | *Biofilm* | Alginate biosynthesis | (*algD*) |
|  | *Biofilm* | Alginate biosynthesis | (*algF*) |
| Iron absorption | HCN synthesis | Biosynthesis and transport of achromobactin | (*acsA*) |
|  | Stimulate ROS | Pyoverdin receptors | (*fpvA*) |
| Biosurfactant | Solubilize Surfactant | Rhamnolipid biosynthesis | (*rhlA*) |
| Toxin | Inhibir a.a. | Phytotoxin phaseotoxin | (*argD*) |
|  |  | Hydrogen cyanide production | (*hcnC*) |

**Table S10.** Virulence genes found in SAICEUPSM^T^ strain, using RAST server.

| ***Mycobacterium* virulence operon involved in protein synthesis (SSU ribosomal proteins)** | | | | |
| --- | --- | --- | --- | --- |
| SAICEUPSM^T^ | | | | |
| Function | Strand | Start | Stop | Size |
| SSU ribosomal protein S12p (S23e) Rv0682 | + | 16526 | 16897 | 372 |
| SSU ribosomal protein S7p (S5e) Rv0683 | + | 17005 | 17478 | 474 |
| Translation elongation factor G Rv0684 | + | 17509 | 19614 | 2106 |
| Translation elongation factor Tu Rv0685 | + | 2861 | 3715 | 855 |
|  | + | 3712 | 4281 | 570 |
|  | + | 19646 | 20839 | 1194 |
| *Ps. atacamensis* M7D1 | | | | |
| Function | Strand | Start | Stop | Size |
| SSU ribosomal protein S12p (S23e) Rv0682 | - | 320656 | 320285 | 372 |
| SSU ribosomal protein S7p (S5e) Rv0683 | + | 17007 | 17480 | 474 |
| Translation elongation factor G Rv0684 | - | 319673 | 317568 | 2106 |
| Translation elongation factor Tu Rv0685 | - | 317536 | 316343 | 1194 |
|  | - | 334094 | 332901 | 1194 |
| ***Mycobacterium* virulence operon involved in DNA transcription** | | | | |
| SAICEUPSM^T^ | | | | |
| DNA-directed RNA polymerase beta subunit (EC 2.7.7.6) Rv0667 | + | 7972 | 12045 | 4074 |
| DNA-directed RNA polymerase beta' subunit (EC 2.7.7.6) Rv0668 | + | 12110 | 16309 | 4200 |
| *Ps. atacamensis* M7D1 | | | | |
| DNA-directed RNA polymerase beta subunit (EC 2.7.7.6) Rv0667 | - | 329210 | 325137 | 4074 |
| DNA-directed RNA polymerase beta' subunit (EC 2.7.7.6) Rv0668 | + | 12112 | 16311 | 4200 |
| **Mycobacterium virulence operon possibly involved in quinolinate biosynthesis** | | | | |
| SAICEUPSM^T^ | | | | |
| Quinolinate synthetase (EC 2.5.1.72) Rv1594 | - | 506564 | 505506 | 1059 |
| L-aspartate oxidase (EC 1.4.3.16) Rv1595 | - | 435416 | 433800 | 1617 |
| Quinolinate phosphoribosyltransferase [decarboxylating] (EC 2.4.2.19) Rv1596 | - | 117187 | 116339 | 849 |
| *Ps. atacamensis* M7D1 | | | | |
| Quinolinate synthetase (EC 2.5.1.72) Rv1594 | + | 441478 | 442536 | 1059 |
| L-aspartate oxidase (EC 1.4.3.16) Rv1595 | - | 732679 | 731063 | 1617 |
| Quinolinate phosphoribosyltransferase [decarboxylating] (EC 2.4.2.19) Rv1596 | - | 102645 | 101797 | 849 |
| **Mycobacterium virulence operon protein synthesis (LSU ribosomal proteins)** | | | | |
| SAICEUPSM^T^ | | | | |
| Translation initiation factor 3 Rv1641 | + | 244824 | 245357 | 534 |
| LSU ribosomal protein L35p Rv1642 | + | 245418 | 245612 | 195 |
| LSU ribosomal protein L20p Rv1643 | + | 245640 | 245996 | 357 |
| *Ps. atacamensis* M7D1 | | | | |
| Translation initiation factor 3 Rv1641 | + | 244469 | 245002 | 534 |
| LSU ribosomal protein L35p Rv1642 | + | 245063 | 245257 | 195 |
| LSU ribosomal protein L20p Rv1643 | + | 245640 | 245996 | 357 |

**Table S11.** Plant growth promotion genes in SAICEU98^T^, directed search.

| Description of the activity | Function | Genetic annotation | Gene Names |
| --- | --- | --- | --- |
| Plant hormones | Auxin biosynthesis | Threonylcarbamoyladenosine biosynthesis protein | (*tsaB*) |
|  | Auxin biosynthesis | Threonylcarbamoyl-AMP synthase | (*tsaC*) |
|  | Auxin biosynthesis | tRNA N6-adenosine threonylcarbamoyltransferase | (*tsaD*) |
|  | Auxin biosynthesis | Threonylcarbamoyladenosine biosynthesis protein | (*tsaE*) |
| Sulfur Metabolism | Cyanate hydrolysis |  | (*cysA*) |
|  | Cyanate hydrolysis | HTH-like transcriptional regulator | (*cysB*) |
|  | Cyanate hydrolysis | Sulfate adenylyltransferase subunit 2 | (*cysD*) |
|  | Cyanate hydrolysis | Serine O-acetyltransferase | (*cysE*) |
|  | Cyanate hydrolysis | Phosphoadenosine phosphosulfate reductase | (*cysH*) |
|  | Cyanate hydrolysis |  | (*cysM*) |
|  | Cyanate hydrolysis | 3'(2'),5'-bisphosphate nucleotidase | (*cysQ*) |
|  | Cyanate hydrolysis | Cysteine ​​- tRNA ligase | (*cynS*) |
|  | Cyanate hydrolysis | Sulfate transporter | (*cysZ*) |
| Nitrogen Metabolism | Nitrosative stress | Anaerobic regulator of nitric oxide reductase transcription. | (*norR*) |
|  | Catalyzes cyanate reaction. | Cyanate hydratase | (*cynR*) |
|  | Catalyzes cyanate reaction. | Cyanate hydratase | (*cynS*) |
| Phosphorus metabolism | Phosphate metabolism | Phosphate transport. ATP binding protein | (*pstB*) |
|  | Phosphate metabolism | Phosphate transport system. permease protein. | (*pstA*) |
|  | Phosphate metabolism |  | (*phoP*) |
|  | Phosphate metabolism | Coenzyme PQQ protein A synthesis | (*pqqA*) |
|  | Phosphate metabolism | Coenzyme PQQ synthesis protein B | (*pqqB*) |
|  | Phosphate metabolism | Pyrroloquinoline-quinone synthase | (*pqqC*) |
|  | Phosphate metabolism | PqqA binding protein | (*pqqD*) |
|  | Phosphate metabolism | Phosphonates importing ATP binding protein | (*phnC*) |
|  | Phosphate metabolism | ABC phosphonate transporter, protein permease | (*phnE*) |
|  | Phosphate metabolism | Ribose 1,5-bisphosphate phosphokinase PhnN | (*phnN*) |
|  | Phosphate metabolism | 2-aminoethylphosphonate - pyruvate transaminase | (*phnW*) |
|  | Phosphate metabolism | Phosphonoacetaldehyde hydrolase | (*phnX*) |
| Protein metabolism | Chaperone | Heat shock protein | (*GrpE*) |
|  | Chaperone | Heat shock protein | (*GroE*) |
|  | Chaperone | Heat shock protein | (*GroS*) |
|  | Chaperone | Heat shock protein | (*GroL*) |
|  | Chaperone | Heat shock protein | (*CspA*) |
| ACC desaminases | ACC desaminases | 1-aminocyclopropane-1-carboxylate deaminase | (*acdS*) |
| Induced systemic resistance | Siderophore uptake | Ferripioverdin receptor | (*fpvA*) |
| Osmoprotectors | Glycine-Beatin production. | Sarcosine oxidase, ß subunit | (*soxB*) |
